# Impact of 25-Hydroxy Vitamin D on White Matter Hyperintensity in Elderly Patients: A Systematic Review and Meta-Analysis

**DOI:** 10.3389/fneur.2021.721427

**Published:** 2022-01-14

**Authors:** Yilei Zhao, Jingfeng Xu, Zhan Feng, Jincheng Wang

**Affiliations:** First Affiliated Hospital, School of Medicine, Zhejiang University, Hangzhou, China

**Keywords:** white matter abnormality, magnetic resonance imaging, vitamin D deficiency, odds ratios, systematic review, meta-analysis

## Abstract

Some studies show that low serum vitamin D levels are associated with white matter hyperintensity (WMH), while other studies report no association. This meta-analysis aimed to investigate the presence of an association between serum 25-hydroxy vitamin D [25(OH)D] levels and WMH. PubMed, Embase, the Cochrane Library, CNKI, WANFANG, and VIP were searched for available papers published up to December 2020. The outcomes were the odds ratios (ORs) with 95% confidence intervals (CIs) for the association between different vitamin D statuses and WMH. All meta-analyses were performed using a random-effects model. Five studies (4393 patients) were included. Compared with sufficient 25(OH)D levels, 25(OH)D deficiency was not associated with WMH (OR = 1.67, 95%CI: 0.92–3.04; I^2^ = 70.2%, *P*_heterogeneity_ = 0.009), nor was 25(OH)D insufficiency (OR = 1.21, 95%CI: 0.89–1.65; I^2^ = 48.1%, *P*_heterogeneity_ = 0.103). A decrease of 25 nmol/L in 25(OH)D levels was associated with WMH (OR = 1.83, 95%CI: 1.34-2.49; I^2^ = 0%, *P*_heterogeneity_= 0.512). The sensitivity analyses showed that the results were robust. 25(OH)D deficiency and insufficiency are not associated with WMH. A decrease of 25 nmol/L in 25(OH)D levels was associated with WMH, but this result will have to be confirmed. Prospective trials, both cross-sectional and longitudinal, are necessary to examine the association between 25(OH)D levels and WMH.

## Introduction

Neurodegenerative diseases are characterized by protein aggregation and inclusion body formation. Most neurodegenerative diseases appear in older people (1, 2). Traditionally, these diseases had to be diagnosed by post-mortem examinations, but recent advances in neuroimaging now allow us to infer the diagnosis of neurodegenerative diseases early in the course of the disease (3). These new techniques involve magnetic resonance imaging (MRI), single-photon emission computed tomography (SPECT), positron emission tomography (PET), protein-specific imaging, and scintigraphy (3). Nevertheless, neuroimaging can also reveal non-specific lesions and signals, and the increased use of these imaging methods led to an increase in the incidence of such lesions (3, 4). Indeed, the aging brain is characterized by a non-specific increase in white matter hyperintensity (WMH), called leukoaraiosis (5–11). WMHlesions are easily visualized on MRI either in the deep white matter (D-WMH) or in contact with lateral cerebral ventricles (periventricularWMH, P-WMH) (5–11). The clinical relevance of both D-WMH and P-WMH is related to the disruption of cortico-subcortical white matter tracts that connect important cognitive regions of the brain (12), making them risk factors associated with cognitive decline (5–7).

Recent prospective studies have found that low serum vitamin D levels are associated with WMH (13–17). It was hypothesized that the age-related decrease in vitamin D level could explain, at least in part, the WMH observed in older adults (15, 18–20). Hypovitaminosis D is a common finding in the elderly (21, 22). Epidemiological studies have reported that older adults with hypovitaminosis D have more frequent and more severe cognitive disturbances than those with appropriate blood levels of vitamin D (23–25). Epidemiological and clinical studies suggest that low 25-hydroxy vitamin D [25(OH)D] levels are associated with higher risks of large-vessel diseases such as myocardial infarction, ischemic stroke, and carotid atherosclerosis (26–31). Consistently, vitamin D is active in the brain and can reverse some age-related brain changes (32). Several actions are described, including participation in neurophysiology through the genetic regulation of neurotransmitters, neurotrophins, and dendritic growth (33–35) and neuroprotective effects based on antioxidant and anti-inflammatory properties (32–34, 36).

On the other hand, some studies suggested that vitamin D levels are not associated with WMH (37, 38). Many of the available studies are characterized by relatively small numbers of individuals, limiting the statistical power for observing differences. In addition, these studies were limited to specific populations, limiting the generalizability of the results. Some studies also included patients with WMH concomitant with other neurological diseases that could influence the associations, such as Alzheimer's disease (39), stroke (17), and dementia (13). In this situation, in view of the lack of large-scale population studies, the meta-analysis methodology can be used to obtain general associations that might help determine a general relationship between 25(OH)D levels and WMH. No previous meta-analysis examined the association between 25(OH)D levels and the presence of WMH. Indeed, 25(OH)D is viewed as a superior biomarker for assessing the vitamin D status (28, 40, 41).

Therefore, this systematic review and meta-analysis aimed to investigate the presence of an association between serum 25(OH)D levels and WMH. The results could help define the role of 25(OH)D as a possible biomarker for WMH.

## Methods

### Literature Search

This systematic review and meta-analysis was performed according to the Preferred Reporting Items for Systematic Reviews and Meta-Analyses (PRISMA) guidelines (42) and the PICO principle (43). PubMed, Embase, the Cochrane Library, CNKI, WANFANG, and VIP were searched for available papers published up to December 2020 using the MeSH terms of “Vitamin D”, “White matter”, “Cerebral disease”, “Leukoaraiosis”, as well as relevant key words, followed by screening based on the eligibility criteria.

### Eligibility Criteria

The eligibility criteria were (1) exposure: 25(OH)D levels, (2) outcome: cerebral disease (WMH by MRI), (3) language: English or Chinese, and (4) outcome: the results were presented as odds ratios (ORs) with 95% confidence intervals (CIs).

### Data Extraction

Study characteristics (authors, year of publication, country, study design, sex, sample size, diagnosis, and cut-off value to define deficiency, insufficient, and sufficient serum 25(OH)D levels) and outcomes (ORs with 95%CIs at different vitamin D statuses; if several models were reported, the data from the most adjusted models were extracted; using sufficient vitamin D as the reference, the ORs of insufficient vitamin D were extracted) were extracted by two different investigators (Yilei Zhao and Jingfeng Xu) according to a pre-specified protocol. Discrepancies in the assessments were reappraised by the third investigator.

### Quality of the Evidence

The level of evidence of all articles was assessed independently by two authors (Zhan Feng and Jincheng Wang) according to the Newcastle-Ottawa Scale (NOS) criteria for quality assessment of cohort and case-control studies (44) and the AHRQ criteria for quality assessment of cross-sectional studies (45). Discrepancies in the assessment were resolved by discussion until a consensus was reached.

### Data Synthesis and Statistical Analysis

For studies that categorized the vitamin levels into four groups, the second and third percentile were synthesized and defined as insufficient vitamin levels in the present meta-analysis. All analyses were performed using STATA SE 14.0 (StataCorp, College Station, TX, USA). Statistical heterogeneity among studies was calculated by Cochran's *Q*-test and the I^2^ index. An I^2^ > 50% and *Q*-test *P* < 0.10 indicated high heterogeneity). All meta-analyses were performed using a random-effects model. *P*-values < 0.05 were considered statistically different. The possible publication bias was not assessed using funnel plots and Egger's test because the number of studies included in each quantitative analysis was <10, in which case the funnel plots and Egger's test could yield misleading results (46, 47).

## Results

### Selection of the Studies

**Figure 1:**
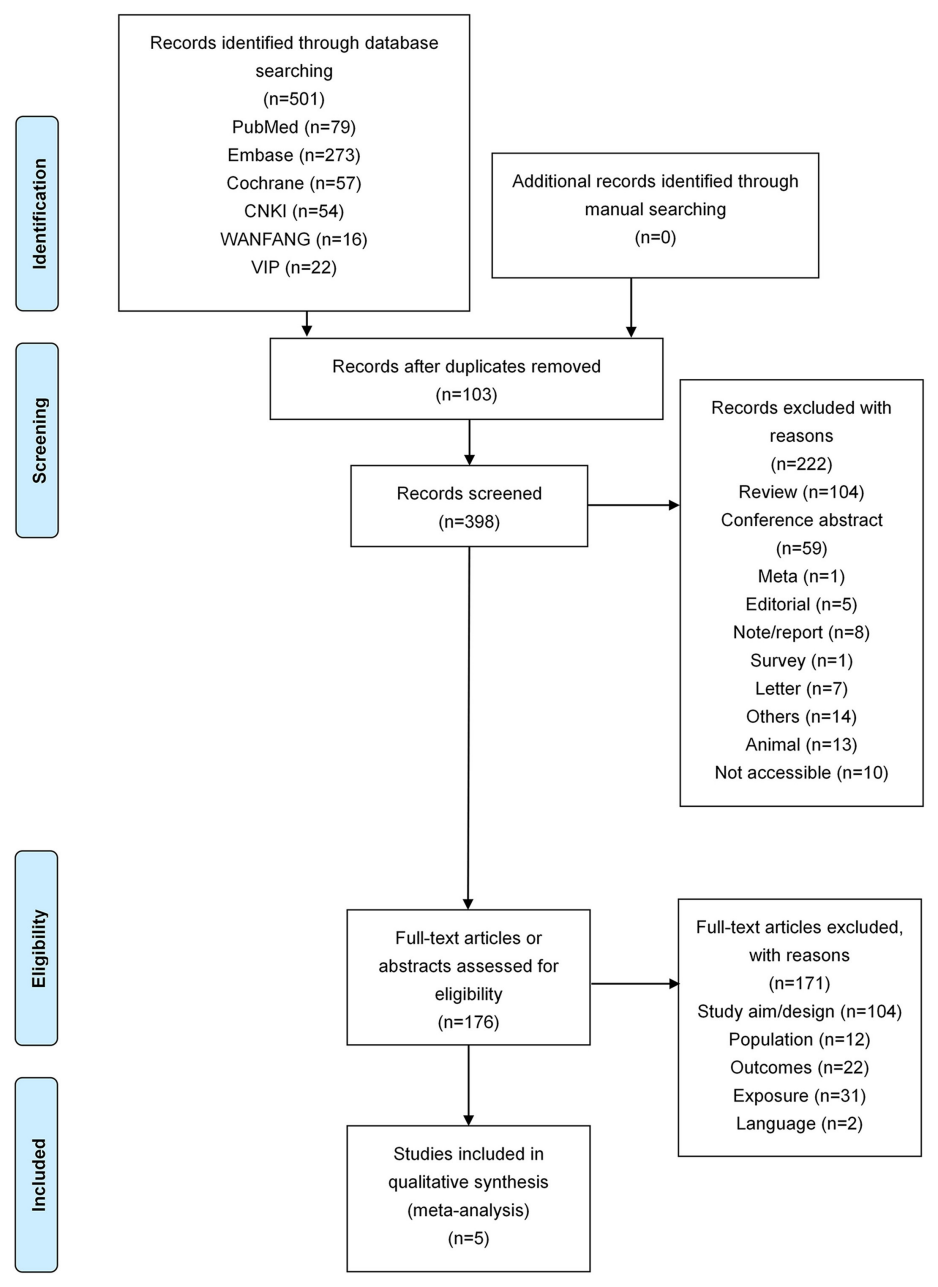
Flow diagram of the study selection process.

Figure 1 presents the study selection process. The initial search yielded 501 records; 103 were removed because they were duplicates, 398 records were screened based on the titles, and 222 were excluded (reviews, *n* = 104; conference abstracts, *n* = 59; meta-analysis, *n* = 1; editorial, *n* = 5; note/report, *n* = 8; survey, *n* = 1; letter, *n* = 7; others, *n* = 14; animal, *n* = 13; not accessible, *n* = 10). Then, 176 full-text articles or abstracts were assessed for eligibility, and 171 were excluded (study aim/design, *n* = 104; population, *n* = 12; outcomes, *n* = 22; exposure, *n* = 31; language, *n* = 2).

Table 1 presents the five included studies (4,393 patients) (16, 17, 37, 38, 48). Two studies were from the United States of America (37, 38), one from Korea (16), and two from China (17, 48). The mean/median age was 62 to 73.6±4.5 years. The percentage of males was 30.8–59.2%. According to the NOS, the case-control study (48) scored 6 points (Supplementary Table 1a), two cohort studies (17, 38) scored 7 points, and one cohort study (37) scored 9 points (Supplementary Table 1b). According to AHRQ criteria, the cross-sectional study (16) scored 7 points (Supplementary Table 1c).

**Table 1:** Literature search and characteristics of the included studies.

**Study**	**Design**	**Country**	**Diagnosis**	***n***	**Age (years, mean or median)**	**Sex, male (%)**	**Serum 25(OH)D levels**			**Serum 25(OH)D level measurement**	**Coefficients of variation**		**MRI**
							**Deficient**	**Insufficient**	**Sufficient**		**Intra**	**Interassay**	
Feng et al. (17)	Prospective cohort study	China	First-ever minor ischemic stroke or transient ischemic attack	234	64.8	56.8	/	/	/	Chemiluminescent immunoassay	1.8%-3.2%	2.2%-3.5%	3.0-T MRI (MAGNETOM Trio 3.0-T, Siemens, Amberg, Germany)
Littlejohns et al. (38)	Prospective cohort study	USA	/	1658	73.6 ± 4.5	30.8	<25 nmol/L	≥25-50 nmol/L	≥50 nmol/L	Liquid chromatography and tandem mass spectrometry (LC-MS) on a Waters Quattro micro mass spectrometer	/	<3.4%	1.5-T/O.35-T MRI
Michos et al. (37)	Prospective cohort study	USA	Cardiovascular disease	1622	62	40.4	<50 nmol/L	50 nmol/L, and <75 nmol/L	≥75 nmol/L	Liquid chromatography–tandem mass spectrometry	/	6.20%	/
Chung et al. (16)	Cross-sectional study	Korea	Acute ischemic stroke or transient ischemic attack	759	68 ± 13	/	≤ 25 nmol/L	>25 nmol/L and ≤ 50 nmol/L	>50 nmol/L	Chemiluminescent immunoassay	5.10%	4.40%	3.0-T MR unit (Avanto, Philips, Eindhoven, The Netherlands
Ma et al. (48)	Case-control study	China	White matter lesion	120	72.16 ± 3.71	59.2	<50 nmol/L	50-75 nmol/L	≥75 nmol/L	/	/	/	/

### Association Between 25(OH)D and WMH

**Figure 2:**
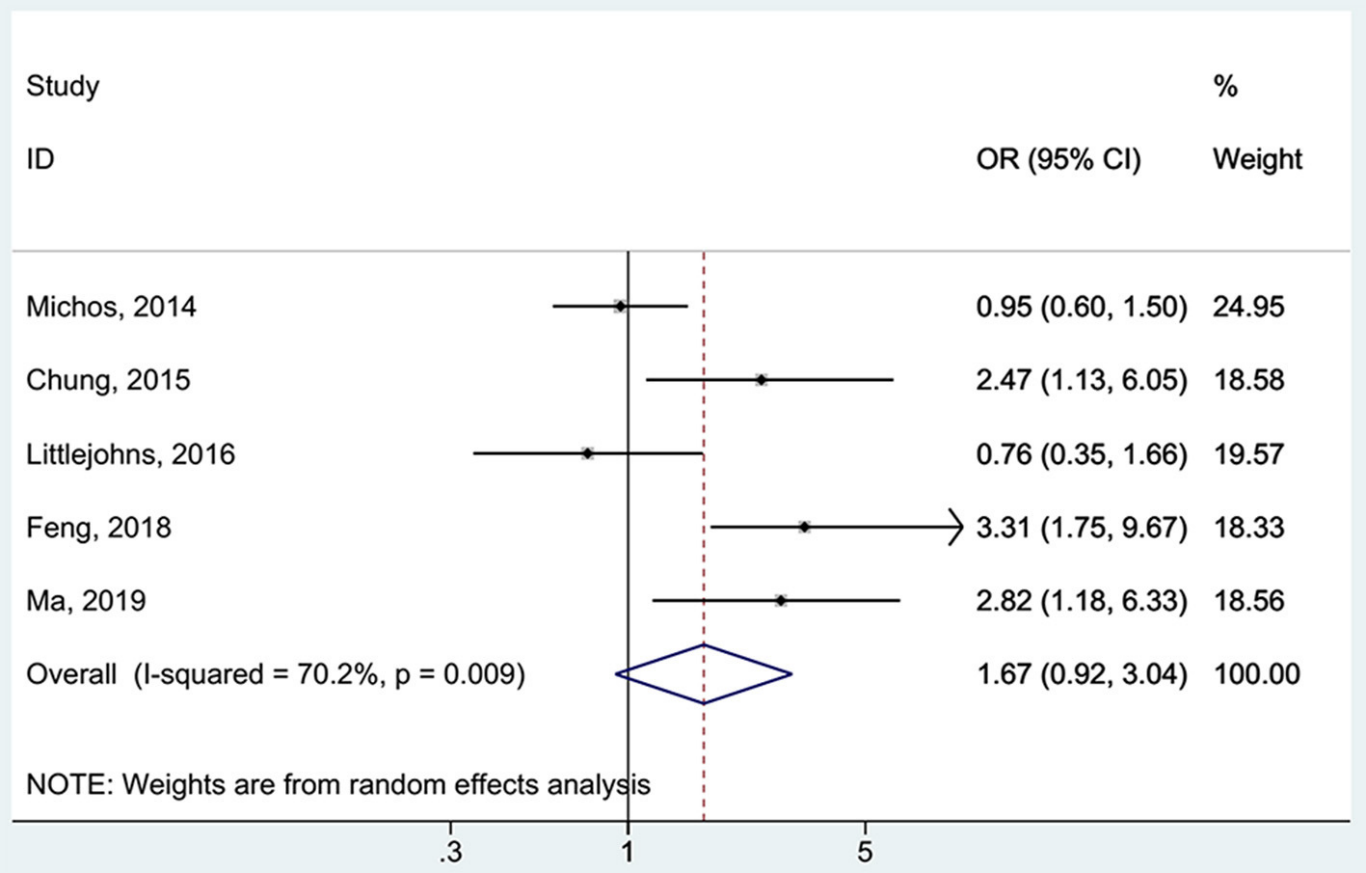
Forest plot of deficient vs. sufficient vitamin D level. % Weight represents the contribution of the sample size of each study to the total number of patients included in the analysis.

**Figure 3:**
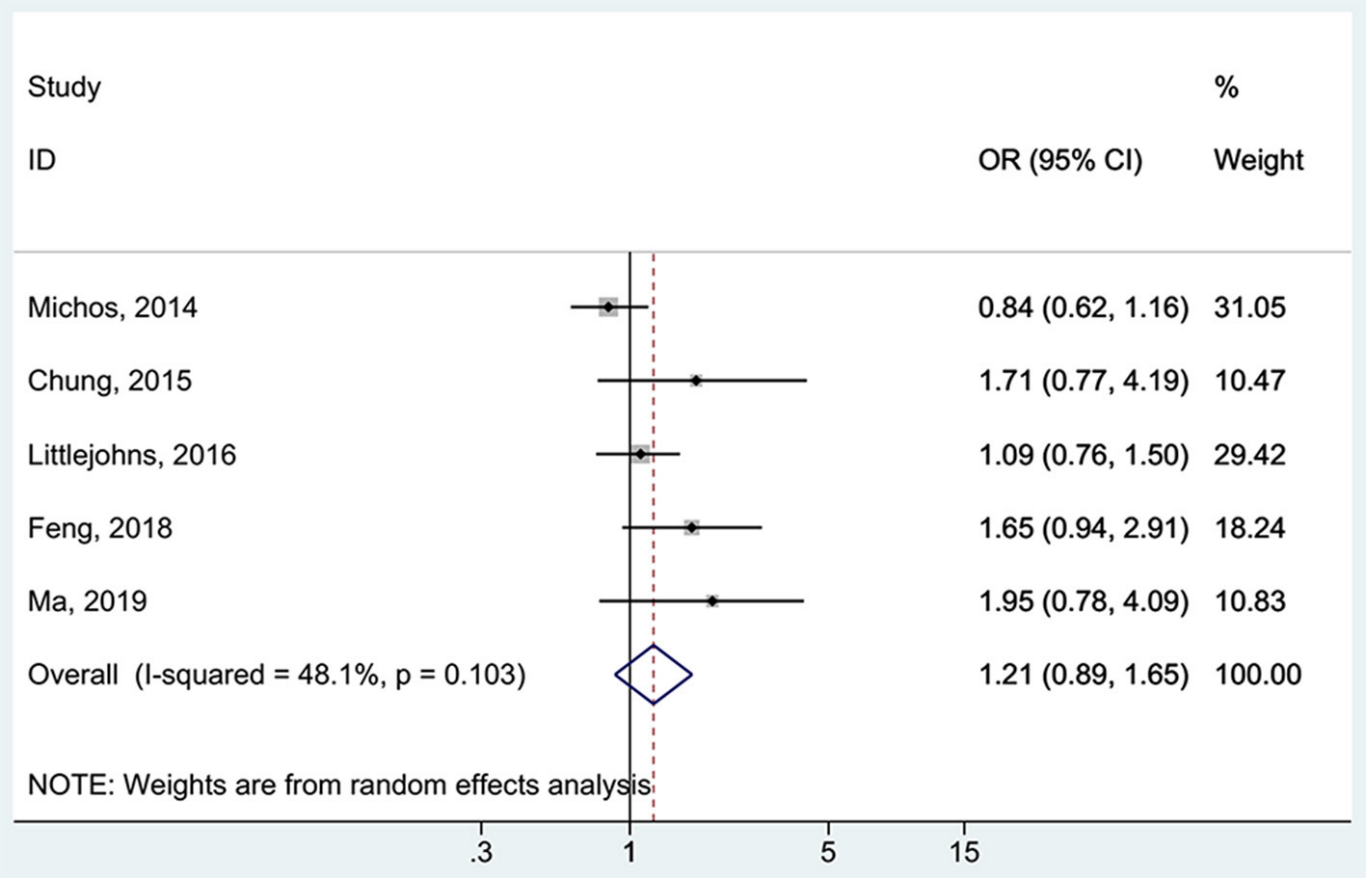
Forest plot of insufficient vs. sufficient vitamin D level. % Weight represents the contribution of the sample size of each study to the total number of patients included in the meta-analysis.

**Figure 4:**
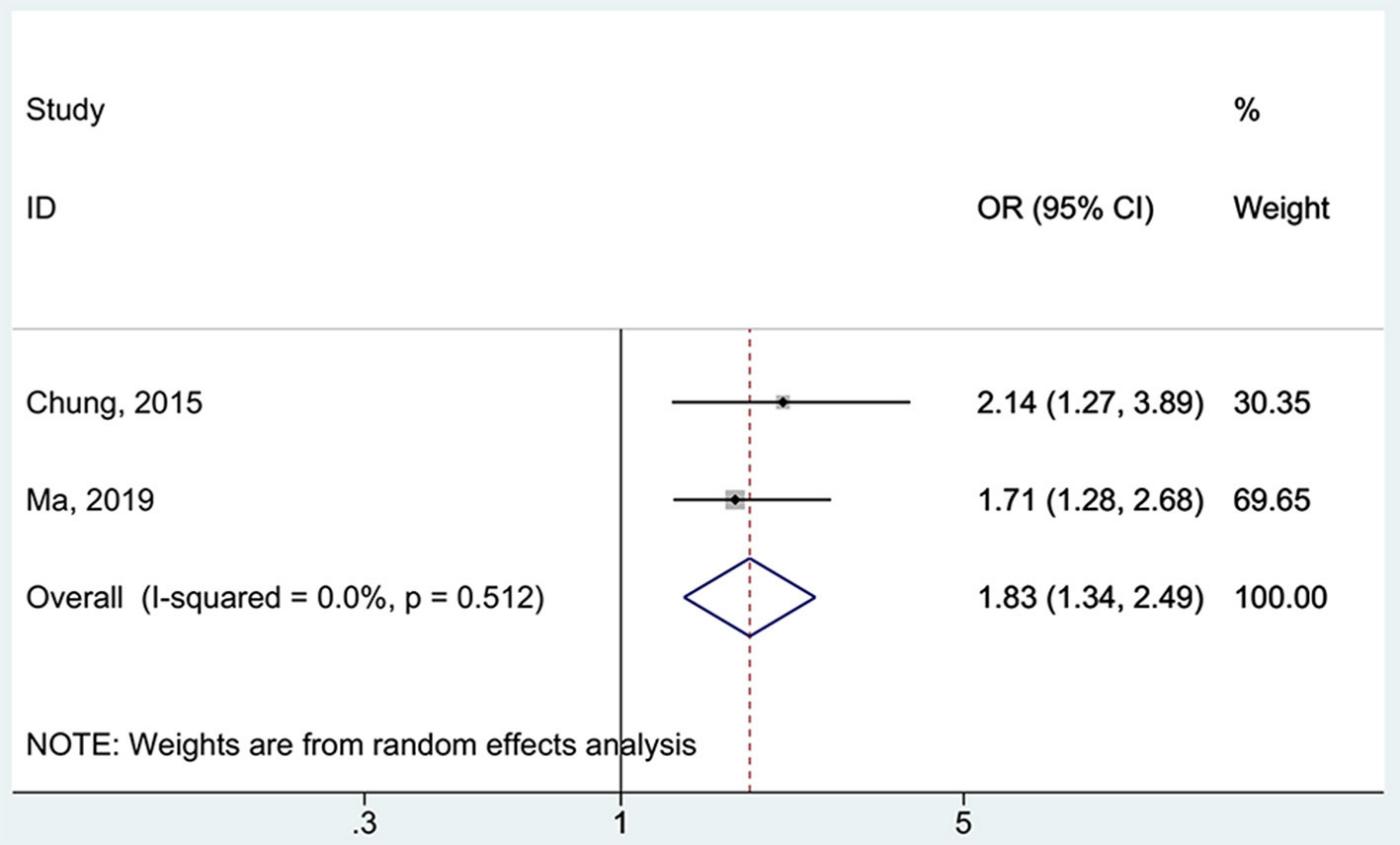
Forest plot of vitamin D decreased by 25 nmol/L. % Weight represents the contribution of the sample size of each study to the total number of patients included in the meta-analysis.

All five studies (16, 17, 37, 38, 48) examined the association between 25(OH)D deficiency/insufficiency and WMH. Compared with the patients with sufficient 25(OH)D levels, those with 25(OH)D deficiency did not show a higher frequency of WMH (OR = 1.67, 95%CI: 0.92–3.04; I^2^ = 70.2%, P_heterogeneity_ = 0.009) (Figure 2). The same was observed for the patients with 25(OH)D insufficiency (OR = 1.21, 95%CI: 0.89–1.65; I^2^ = 48.1%, P_heterogeneity_ = 0.103) (Figure 3). Using regression analyses, two studies (16, 48) showed that a decrease of 25 nmol/L in the absolute levels of 25(OH)D was associated with WMH lesions (OR = 1.83, 95%CI: 1.34–2.49; I^2^ = 0%, P_heterogeneity_= 0.512) (Figure 4).

### Sensitivity Analyses

**Figure 5:**
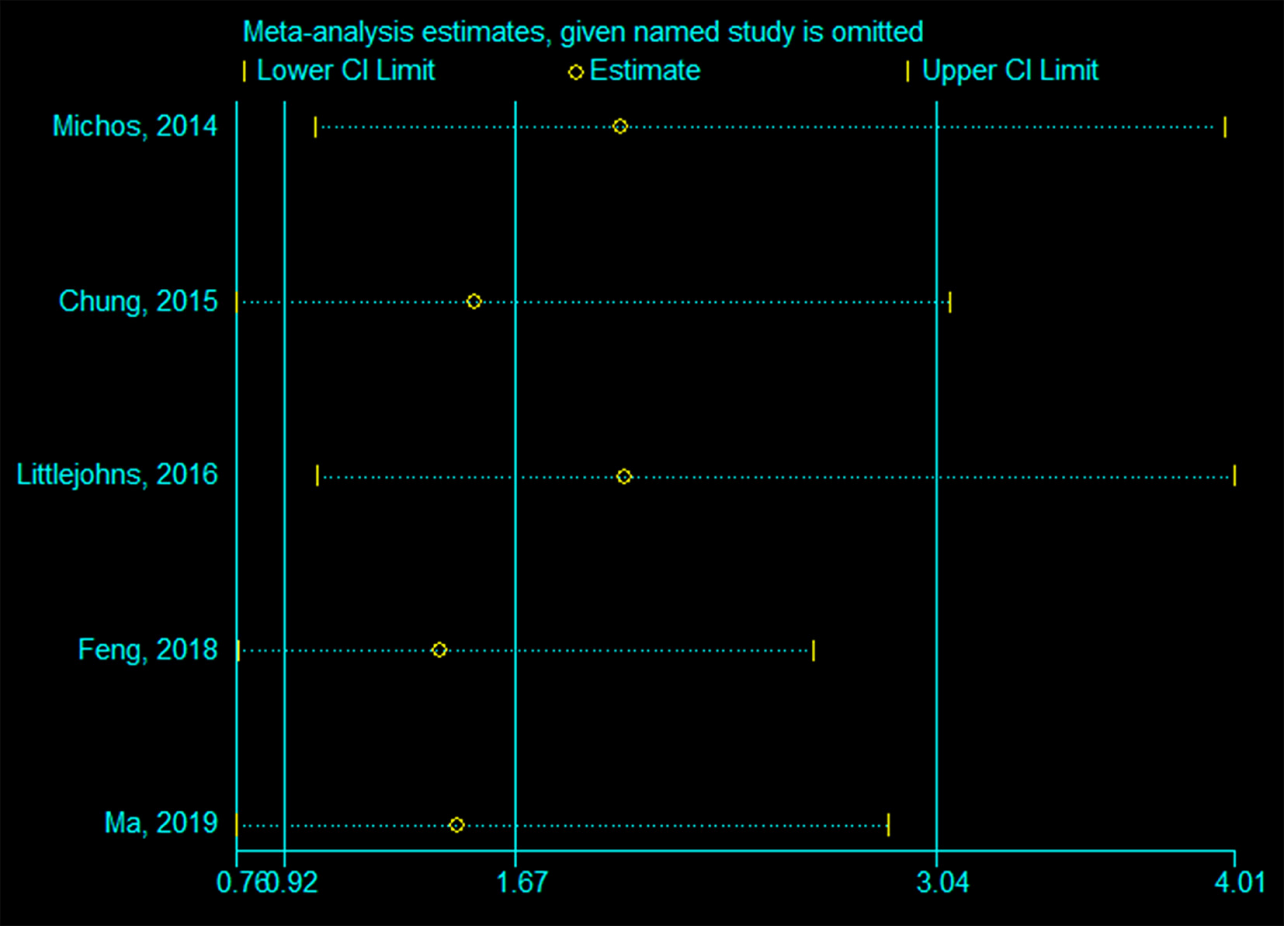
Sensitivity analysis of deficient vs. sufficient vitamin D level.

**Figure 6:**
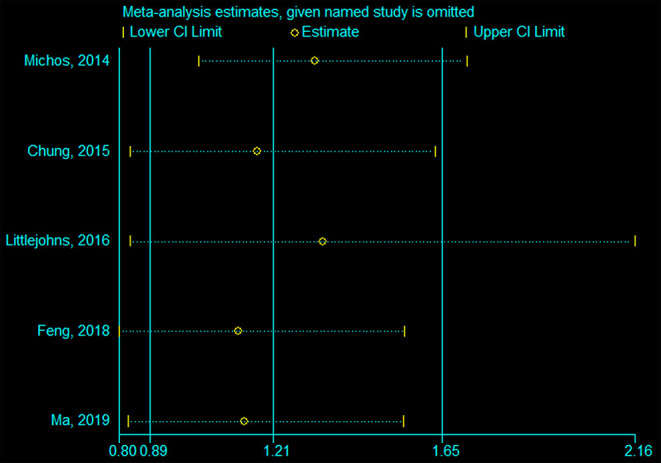
Sensitivity analysis of insufficient vs. sufficient vitamin D level.

Figures 5, 6 indicate that the analyses were robust. The sequential exclusion of each study did not influence the results significantly.

## Discussion

Evidence suggests that low serum vitamin D levels are associated with WMH (13–20), but conflicting results are reported among studies (37, 38). Therefore, this study aimed to investigate the presence of an association between serum 25-hydroxy vitamin D [25(OH)D] levels and WMH. The results showed that 25(OH)D deficiency and insufficiency are not associated with WMH, but a decrease of 25 nmol/L in 25(OH)D levels were associated with WMH.

WMH has retained attention because of its association with cognitive decline in older adults (5–7). Vitamin D levels are associated with neurological conditions involving lower cognitive abilities levels (23–25). Thus, some authors suggested that there might be an association between vitamin D levels and WMH (15, 18–20). This association is mechanistically possible since WMH on MRI represents brain areas where the cortico-subcortical white matter tracts are disrupted (12) and because vitamin D is known to have several neuroprotective effects (32–36). The association between low vitamin D levels and WMH has been suggested by several studies (13–20), but not all of them could be included in the present meta-analysis because of the selection criteria and because some of them did not measure 25(OH)D, which is considered the most reliable marker for vitamin D metabolism (28, 40, 41). Still, they support the neuroprotective effects of vitamin D. On the other hand, other studies do not support this association (37, 38). The present meta-analysis also supports that 25(OH)D deficiency and insufficiency are not associated with WMH. This observation might be because vitamin D is only one of many factors that affect WMH development. Still, only five studies could be included, and the highly negative results of Michos et al. (37) and Littlejohns et al. (38) might drive the lack of association. In addition, because there were <10 studies included, publication bias could not be analyzed (47). Future studies should examine these other factors as well.

Significant heterogeneity was observed in this meta-analysis and could be explained by the characteristics of the included studies. In the study by Feng et al. (17), 234 patients with first-ever minor ischemic stroke or transient ischemic attacks (TIA) and who underwent MRI were included. 25(OH)D levels were measured using an immunoassay. They concluded that 25(OH)D levels were associated with WMH in patients with minor brain ischemia. That study, of course, suffers from a selection bias. Chung et al. (16) examined the 25(OH)D levels (by immunoassay) of 759 patients who underwent MRI for ischemic stroke or TIA. They showed that 25(OH)D levels were inversely associated with WMH. Ma et al. (48) examined the association between 25(OH)D levels and WMH in 163 patients with lacunar infarction and 158 healthy volunteers. They showed that the 25(OH)D levels were associated with lacunar infarction and with the presence of WMH. In the longitudinal study by Michos et al. (37), participants without neurological conditions at baseline underwent MRI (*n* = 1622) and a second MRI 10 years later (*n* = 888). That study was based on the Atherosclerosis Risk in Communities (ARIC) Brain MRI study. The 25(OH)D levels were measured only at baseline, and this cross-sectional measurement was not associated with WMH development. Littlejohns et al. (38) also performed a longitudinal study, based on the US-based Cardiovascular Health Study, in which 1,658 participants aged >65 years and free of any cognitive-related neurological condition at baseline (in 1992–1993) were followed using MRI for a mean follow-up of 5.0 years. Hence, using a longitudinal design, they showed that 25(OH)D levels were not associated with WMH development in elderly patients. The measurement of 25(OH)D levels by mass spectrometry and liquid chromatography-tandem mass spectrometry, as in the two longitudinal studies (37, 38), is far more sensitive and precise than immunoassays but more expensive and requires more equipment and technical expertise (49, 50). Unfortunately, in the two longitudinal studies, only baseline 25(OH)D levels were measured, explaining the lack of association with WMH. Future longitudinal studies should perform multiple measurements of 25(OH)D in time. On the other hand, three studies (16, 17, 48) showed associations between 25(OH)D levels and WMH were performed in patients who underwent MRI because of stroke or TIA, resulting in selection bias and limiting the generalizability of the results.

Differences in vitamin D levels among countries and ethnicities could indeed influence the associations observed among the various studies and contribute to heterogeneity. In the USA population, Parva et al. (51) reported that low levels of vitamin D were observed in Hispanics and African-Americans. Wei et al. (52) reported that the prevalence of vitamin D deficiency was high in China and in ethnic minorities in the USA. The prevalence of vitamin D deficiency is higher among individuals of African origin and living at high latitudes (53). There are also large differences in vitamin D deficiency prevalence among European and Middle East countries (54).

This meta-analysis has limitations. Because of the strict eligibility criteria, the number of studies that could be included was small. All five studies included together 4,393 patients. Considering that WMH must be diagnosed by MRI, it is a relatively large number of patients. On the other hand, epidemiologically speaking, this number is small, and only three countries are represented. Additional studies are necessary to determine the epidemiological characteristics of WMH in relation to 25(OH)D levels. The present meta-analysis highlights this need. Furthermore, the quality of a meta-analysis is determined by the quality of the studies that match the eligibility criteria, and the quality of the included studies was relatively low. The definitions for vitamin D sufficiency, vitamin D insufficiency, and vitamin D deficiency varied among the studies and the methods for measuring the 25(OH)D levels. In addition, the selection of the patients, as well as the indications for MRI, varied among studies. These factors contributed to the heterogeneity observed among the studies. Finally, many causative relationships can be responsible for the association between WMH progression and cognitive impairment, including 25(OH)D levels and aging, 25(OH)D and impairment of cognitive functions, and white matter change and aging, among others. This meta-analysis was not designed to determine the causal relationships, and several covariates affecting cognitive impairment could not be considered. Future studies will have to examine such relationships more closely.

In conclusion, 25(OH)D deficiency and insufficiency are not associated with WMH. A decrease of 25 nmol/L in 25(OH)D levels was associated with WMH, but this result will have to be confirmed. Prospective trials, both cross-sectional and longitudinal, are necessary to examine the association between 25(OH)D levels and WMH, as well as the causal relationships.

## Data Availability Statement

The original contributions presented in the study are included in the article/Supplementary Material, further inquiries can be directed to the corresponding author/s.

## Author Contributions

All authors designed the systematic review protocol. JX and ZF searched the literature for potentially eligible studies. ZF and JW carried the selection of studies. YZ extracted the information. YZ and JX evaluated the methodology quality. ZF conducted the meta-analysis. YZ wrote this paper.

## Funding

This study was funded by the Zhejiang Natural Science Foundation, Association of Mathematical and Physical Medicine (No. LSY19H180014); Zhejiang Basic Public Welfare Research Project (No. LGF19H220003).

## Conflict of Interest

The authors declare that the research was conducted in the absence of any commercial or financial relationships that could be construed as a potential conflict of interest.

## Publisher's Note

All claims expressed in this article are solely those of the authors and do not necessarily represent those of their affiliated organizations, or those of the publisher, the editors and the reviewers. Any product that may be evaluated in this article, or claim that may be made by its manufacturer, is not guaranteed or endorsed by the publisher.
