# Supplementary material for: Impact of 25-Hydroxy Vitamin D on White Matter Hyperintensity in Elderly Patients: A Systematic Review and Meta-Analysis
Source: Front Neurol. 2022 Jan 14;12:721427. doi: 10.3389/fneur.2021.721427 (PMC8794798; doi:10.3389/fneur.2021.721427)
Supplement: Supplementary file 1 [file Data_Sheet_1.docx]

**Supplementary Table S1a.** NOS criteria for quality of cohort studies

| Study | Representativeness of the exposed cohort | Selection of the non-exposed cohort | Ascertainment of exposure | Demonstration that outcome of interest was not present at the start of the study | Comparability of cohorts based on the design or analysis | Assessment of outcome | Was follow-up long enough for outcomes to occur | Adequacy of follow up of cohorts | Total quality scores |
| --- | --- | --- | --- | --- | --- | --- | --- | --- | --- |
| Feng, 2018 (17) | ☆ | ☆ | ☆ | ☆ | ☆☆ | ☆ | / | / | 7 |
| Littlejohns, 2016 (38) | ☆ | ☆ | ☆ | ☆ | ☆☆ | ☆ | / | / | 7 |
| Michos, 2014 (37) | ☆ | ☆ | ☆ | ☆ | ☆☆ | ☆ | ☆ | ☆ | 9 |

**Supplementary Table S1b.** NOS criteria for quality of case-control study

| Study | Is the case definition adequate? | Representativeness of the cases | Selection of controls | Definition of controls | Comparability of cases and controls based on the design or analysis | Ascertainment of intervention | Same method of ascertainment for cases and controls | Non-response rate | Total quality scores |
| --- | --- | --- | --- | --- | --- | --- | --- | --- | --- |
| Ma, 2019 (48) | ☆ | ☆ | ☆ | ☆ | ☆☆ | / | / | / | 6 |

**Supplementary Table S1c.** AHRQ criteria for quality of cross-sectional study

| Study | Define the source of information | List inclusion and exclusion criteria for exposed and unexposed subjects or refer to previous publications | Indicate time period used for identifying patients | Indicate whether or not subjects were consecutive if not population-based | Indicate if evaluators of subjective components of the study were masked to other aspects of the status of the participants | Describe any assessments undertaken for quality assurance purposes | Explain any patient exclusions from the analysis | Describe how confounding was assessed and/or controlled | If applicable, explain how missing data were handled in the analysis | Summarize patient response rates and completeness of data collection | Clarify what follow-up, if any, was expected and the percentage of patients for which incomplete data or follow-up was obtained | Total quality scores |
| --- | --- | --- | --- | --- | --- | --- | --- | --- | --- | --- | --- | --- |
| Chung, 2019 (16) | ★ | ★ | ★ | ★ | / | ★ | ★ | ★ | / | / | / | 7 |
